# Celastrol‐Loaded Conductive Hydrogel Mitigates Myocardial Ischemia‐Reperfusion Injury and Restores Electrophysiological Function

**DOI:** 10.1002/advs.202524201

**Published:** 2026-07-10

**Authors:** Shixin Wang, Shaojie Chen, Chengzong Li, Hongyi Cheng, Jincheng Jiao, Xiafeng Peng, Yike Zhang, Wei Sun, Feng Zhang, Chang Cui, Minglong Chen

**Affiliations:** ^1^ Department of Cardiology The First Affiliated Hospital with Nanjing Medical University Nanjing P. R. China; ^2^ Department of Cardiology The Affiliated Hospital of Xuzhou Medical University Xuzhou P. R. China; ^3^ Jiangsu Clinical Medicine Research Institute The First Affiliated Hospital with Nanjing Medical University Nanjing P. R. China; ^4^ Department of Cardiology The Affiliated Taizhou People's Hospital of Nanjing Medical University Taizhou School of Clinical Medicine Nanjing Medical University Taizhou P. R. China

**Keywords:** antioxidant, celastrol, conductive hydrogel, drug delivery, myocardial ischemia–reperfusion injury

## Abstract

Myocardial ischemia–reperfusion injury (MIRI) remains an unresolved clinical challenge that severely limits the prognosis of patients undergoing revascularization. Through a phenotype‐guided antioxidant screening workflow, celastrol (CLT) was selected as a bioactive compound for integration into a locally retained conductive hydrogel platform. However, the clinical translation of CLT is limited by poor aqueous solubility and potential systemic toxicity. To overcome these limitations, we developed an injectable F127DA/GelMA/PEDOT:PSS/celastrol hydrogel (FGPC) for the localized and sustained delivery of CLT. The FGPC hydrogel, composed of Gelatin methacryloyl (GelMA), Pluronic F127 diacrylate (F127DA) , and conductive poly(3,4‐ethylenedioxythiophene):poly(styrenesulfonate) (PEDOT:PSS), exhibits favorable mechanical properties, enhanced electrical conductivity, local retention, and controlled drug release. In vitro, FGPC effectively reduced reactive oxygen species (ROS), supported cardiomyocyte structural organization, and enhanced gap‐junction coupling. In a rat MIRI model, local FGPC delivery attenuated acute oxidative stress, suppressed inflammatory activation, and reduced neutrophil extracellular trap formation. Transcriptomic analysis further revealed downregulation of inflammatory pathways, including the IL‐17 signaling pathway and S100a8/S100a9‐related inflammatory mediators. During the chronic repair phase, FGPC improved cardiac function, reduced fibrosis, restored connexin 43 expression, reduced inducible ventricular arrhythmia susceptibility, and promoted angiogenesis without obvious systemic toxicity. This study presents a locally retained conductive hydrogel platform that combines CLT‐mediated microenvironment modulation with electrical support, providing a potential strategy for myocardial repair after reperfusion.

## Graphical abstract

Schematic illustration of the Pluronic F127 diacrylate/gelatin methacryloyl/poly(3,4‐ethylenedioxythiophene):poly(styrenesulfonate)/celastrol hydrogel (FGPC) for localized myocardial ischemia–reperfusion injury treatment. FGPC combines sustained celastrol delivery with PEDOT:PSS‐mediated conductive support to attenuate acute oxidative/inflammatory injury and promote chronic myocardial repair.

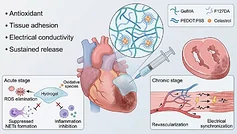

## 1 Introduction

Acute myocardial infarction (AMI) remains a major global health burden, accounting for millions of deaths annually, with approximately two million cases reported in China each year [1, 2]. Early reperfusion therapies, including primary percutaneous coronary intervention (PCI) and thrombolysis, are established clinical strategies to restore coronary blood flow, limit infarct size, and significantly reduce both short‐ and long‐term mortality [3, 4, 5]. Nevertheless, despite continuous advances in reperfusion therapy, the in‐hospital mortality rate of patients with ST‐segment elevation myocardial infarction (STEMI) remains between 2.2% and 6.1%, partly because reperfusion itself can trigger myocardial ischemia–reperfusion injury (MIRI) [6]. MIRI initiates a series of pathological events, and the secondary myocardial injury caused by reperfusion may further aggravate the initial ischemic insult [7, 8, 9]. Given the limited regenerative capacity of adult cardiomyocytes, post‐infarction repair is largely characterized by fibrotic scar formation. This non‐contractile and poorly conductive scar tissue contributes to adverse ventricular remodeling, impaired cardiac function, and increased arrhythmogenic susceptibility [10].

During the early phase of MIRI, damage‐associated molecular patterns (DAMPs) and inflammatory mediators promote neutrophil recruitment to the injured myocardium. In the oxidative microenvironment, reactive oxygen species (ROS) activate neutrophils and promote the release of proinflammatory cytokines, chemokines, proteases, and neutrophil extracellular traps (NETs), thereby amplifying cardiomyocyte death and tissue injury [11, 12, 13, 14, 15]. Current therapeutic options for MIRI remain limited [16]. Systemic pharmacological interventions are constrained by insufficient accumulation in the ischemic myocardium, rapid systemic metabolism, and off‐target effects [17]. For instance, although antioxidants theoretically scavenge ROS, their lack of cardiac specificity and limited local retention frequently reduce their therapeutic efficacy in the reperfused myocardium [18]. Together, these limitations highlight the need for therapeutic strategies that can achieve localized and sustained modulation of the injured myocardial microenvironment, attenuate early oxidative and inflammatory injury, and reduce subsequent adverse remodeling after reperfusion [19].

Celastrol (CLT), a bioactive triterpenoid derived from *Tripterygium wilfordii* Hook.f., has demonstrated therapeutic potential in inflammatory and autoimmune diseases, including rheumatoid arthritis [20, 21, 22]. CLT has been reported to regulate inflammatory signaling and oxidative stress in multiple disease contexts [23, 24]. CLT can also attenuate inflammation and oxidative stress through signaling pathways such as the AMPKα/Nrf2/TXNIP pathway [25]. Previous studies have suggested that CLT may protect against ischemia/reperfusion‐related injury within an appropriate concentration range [26]. However, its broader therapeutic application is limited by poor aqueous solubility, rapid clearance, and dose‐dependent systemic toxicities involving the heart, liver, hematopoietic system, and kidney [27, 28, 29]. Therefore, integrating CLT into a locally retained biomaterial carrier may provide a practical strategy to reshape its in vivo exposure pattern, prolong cardiac retention, and improve its therapeutic applicability in MIRI.

Hydrogels have attracted increasing attention for myocardial repair because of their high water content, tissue‐like mechanical properties, and capacity for localized delivery of therapeutic agents [30, 31, 32]. As injectable or in situ‐forming biomaterials, hydrogels can improve local retention and enable sustained release within the injured myocardium, thereby reducing the limitations of systemic administration [33, 34]. However, conventional hydrogels are generally electrically insulating and may not adequately support the electrical coupling required for functional myocardial repair. Incorporation of conductive components such as poly(3,4‐ethylenedioxythiophene):poly(styrenesulfonate) (PEDOT:PSS) can improve hydrogel conductivity and provide an electroactive microenvironment that supports impulse propagation, cardiomyocyte coupling, and synchronized contraction [35, 36, 37]. Thus, a locally retained conductive hydrogel may simultaneously address two key pathological features of MIRI: acute oxidative/inflammatory injury and chronic structural/electrical remodeling.

Herein, we designed an injectable conductive hydrogel for localized CLT delivery and myocardial microenvironment modulation after MIRI. Gelatin methacryloyl (GelMA) was used as a biocompatible and enzymatically degradable network component, while Pluronic F127 diacrylate (F127DA) was incorporated to improve mechanical resilience and facilitate hydrophobic CLT loading through micellar encapsulation. CLT, a potent anti‐inflammatory and antioxidant agent, was encapsulated within F127DA micelles, yielding an antioxidant micelle–hydrogel composite capable of mitigating oxidative stress. PEDOT:PSS was further introduced to endow the hydrogel with electrical conductivity. The resulting F127DA/GelMA/PEDOT:PSS/CLT hydrogel (FGPC) was designed to provide local retention, sustained CLT release, and conductive microenvironment support. By combining CLT‐mediated antioxidant and anti‐inflammatory effects with PEDOT:PSS‐mediated electrical support, FGPC aims to attenuate acute reperfusion injury while promoting chronic myocardial repair.

To evaluate this strategy, FGPC was locally applied to the epicardial surface of rat hearts immediately after reperfusion in a MIRI model. The therapeutic effects of FGPC were assessed across both the acute injury phase and the chronic repair phase. In the acute phase, we examined oxidative stress, inflammatory activation, NET formation, and transcriptomic changes in the infarct border zone. In the chronic phase, we evaluated cardiac function, fibrosis, gap‐junction remodeling, arrhythmia susceptibility, and neovascularization. Together, this study establishes a locally retained conductive hydrogel platform that integrates sustained CLT delivery with electrical microenvironment support, providing a potential approach for mitigating reperfusion injury and promoting myocardial repair (Figure 1).

**FIGURE 1:**
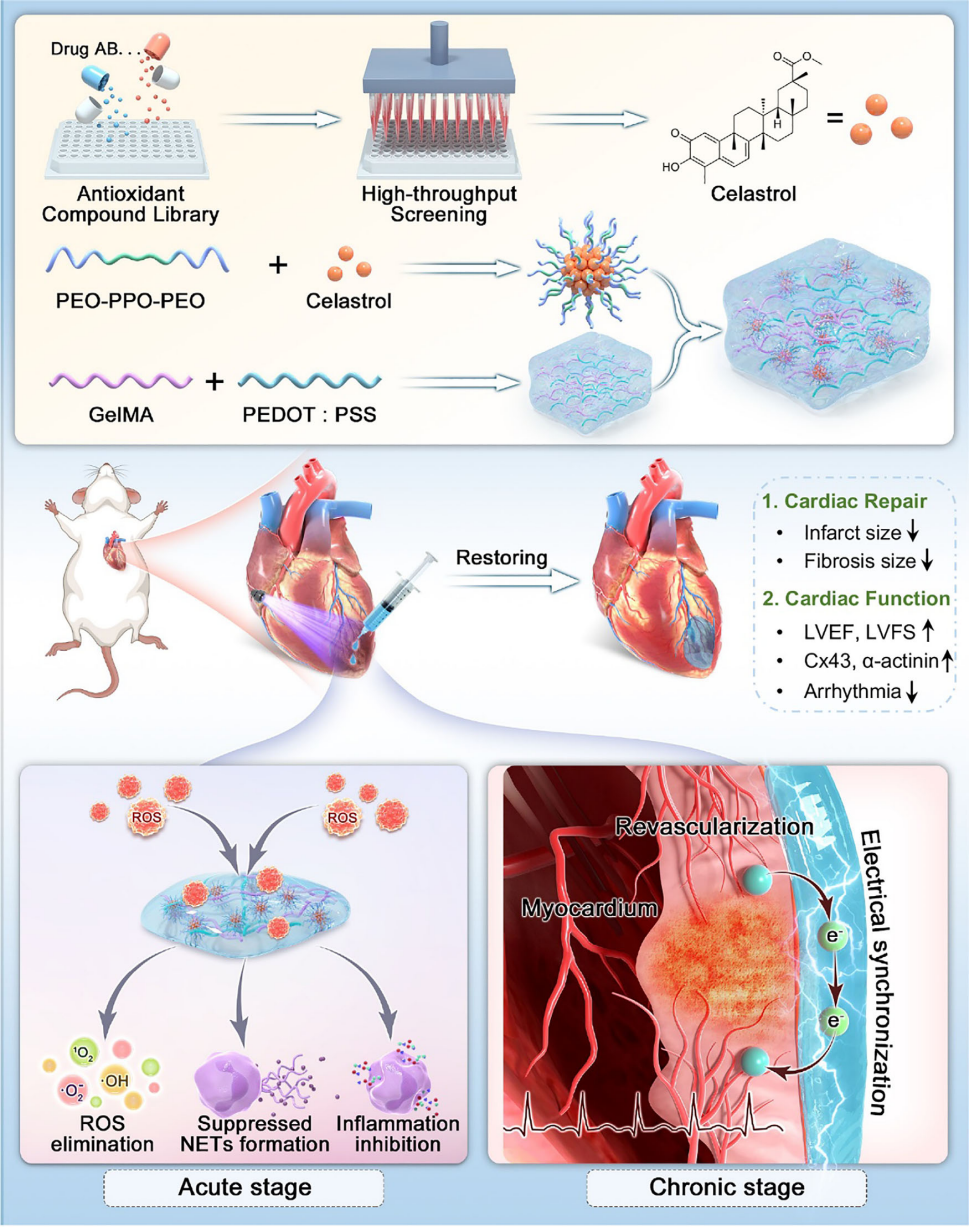
Schematic illustration of FGPC hydrogel construction and its therapeutic effects after myocardial ischemia–reperfusion injury. Celastrol was identified through antioxidant compound screening and incorporated into a conductive F127DA/GelMA/PEDOT:PSS hydrogel to form FGPC. Following local epicardial application to the injured myocardium, FGPC attenuates acute oxidative stress, NET formation, and inflammation, while promoting chronic myocardial repair by enhancing revascularization, electrical synchronization, and cardiac functional recovery.

## 2 Results and Discussion

### 2.1 Antioxidant Screening and Concentration‐Window Evaluation of Celastrol

To identify a suitable bioactive cargo for incorporation into our hydrogel platform, we performed a phenotype‐guided antioxidant screening using the Selleck Antioxidant Compound Library in an H_2_O_2_‐induced oxidative‐stress model in AC16 cells. Cells were pretreated with each compound at 1 or 10 µm, followed by H_2_O_2_ exposure, and intracellular ROS levels were assessed using DCFH‐DA fluorescence (Figure 2a,b). The top‐ranked ROS‐lowering compounds at each concentration were then compared (Figure 2c,d). Among the screened compounds, celastrol (CLT) consistently showed strong ROS‐lowering activity under our experimental conditions and was therefore selected for further validation as a candidate cargo for hydrogel integration. Given the previously reported antioxidant and anti‐inflammatory properties of CLT, we next focused on defining its usable concentration window in the present AC16 oxidative‐stress model [25, 38].

**FIGURE 2:**
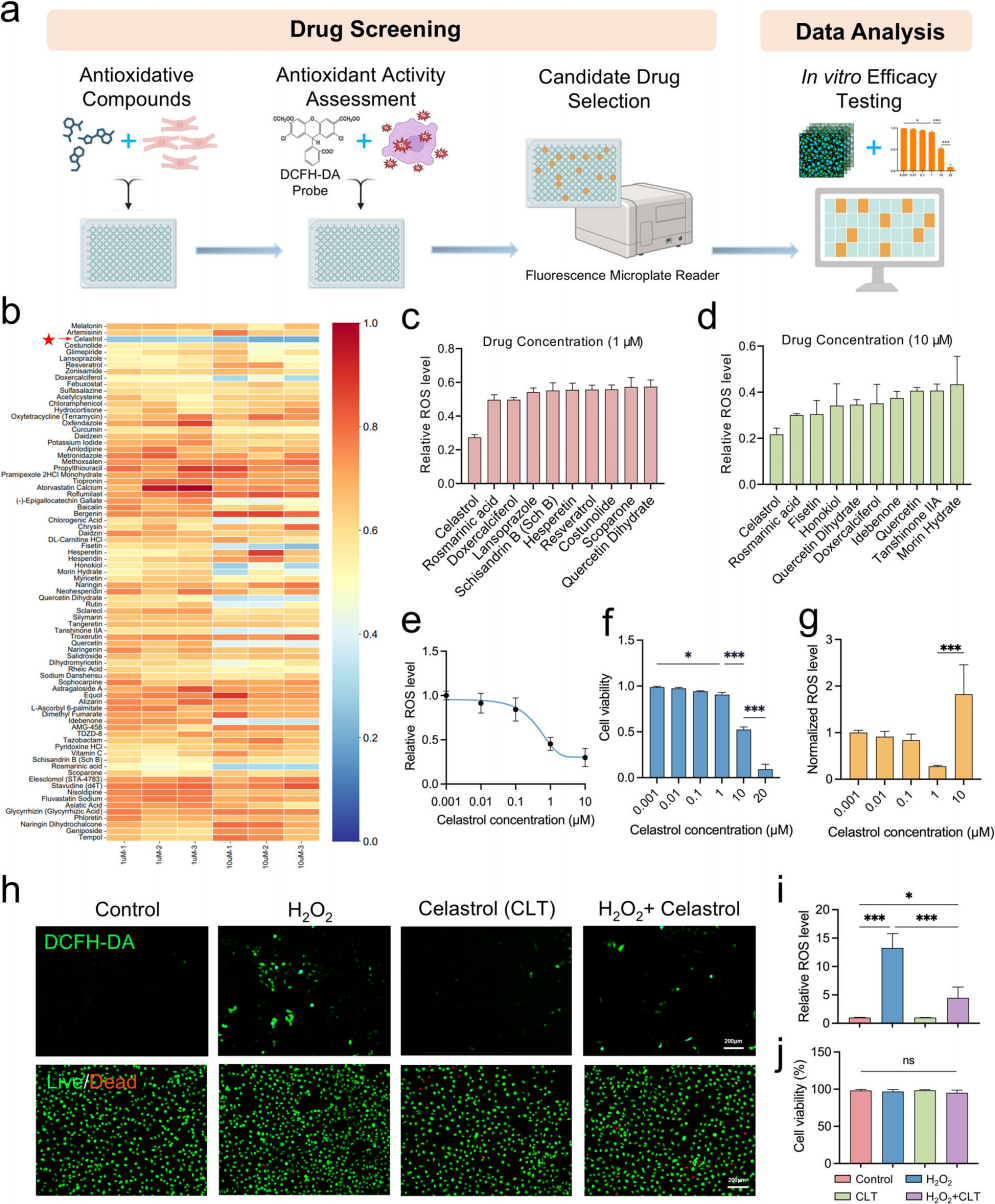
Antioxidant screening and concentration‐window evaluation of celastrol. (a) Schematic illustration of the antioxidant compound screening workflow. A 96‐well antioxidant compound library was screened in AC16 cells under H_2_O_2_‐induced oxidative stress, followed by fluorescence‐based ROS detection and candidate compound ranking. (b) Heatmap showing relative intracellular ROS levels after treatment with library compounds at 1 and 10 µm. The red star indicates celastrol, which was selected from the screening results for further validation (*n* = 3). (c, d) Top‐ranked antioxidant candidates based on ROS reduction at 1 µm (c) and 10 µm (d). (e) Dose‐dependent effect of celastrol on intracellular ROS levels in AC16 cells (*n* = 3). (f) Cell viability of AC16 cells treated with different concentrations of celastrol, as determined by CCK‐8 assay (*n* = 3). (g) ROS levels normalized to cell viability to evaluate antioxidant activity independent of cytotoxicity (*n* = 3). (h) Representative 2′,7′‐dichlorodihydrofluorescein diacetate (DCFH‐DA) fluorescence images and Live/Dead staining images of AC16 cells after different treatments. Green fluorescence in DCFH‐DA staining indicates intracellular ROS; green and red signals in Live/Dead staining indicate live and dead cells, respectively. Scale bars, 200 µm. (i) Quantification of intracellular ROS fluorescence intensity (*n* = 4). (j) Quantification of cell viability (*n* = 4). Data are presented as mean ± SD. Statistical significance was determined by one‐way ANOVA followed by Tukey's post hoc test. (^*^ *p* < 0.05, ^**^ *p* < 0.01, ^***^ *p* < 0.001; ns, not significant.).

To further define the usable concentration window of CLT, we evaluated its ROS‐reducing effect and cytocompatibility over a broad concentration range. CLT showed a concentration‐dependent increase in antioxidant activity (Figure 2e). However, cell viability declined markedly at 10 µm and above, indicating clear concentration‐dependent cytotoxicity (Figure 2f). This finding is consistent with previous reports that celastrol has a narrow therapeutic window in ischemia/reoxygenation‐related settings [26, 39, 40]. After normalization of ROS levels to cell viability, 1 µm CLT showed the most favorable balance between antioxidant efficacy and safety (Figure 2g). Although higher concentrations produced lower raw ROS signals, the viability‐corrected ROS levels were not further improved, suggesting that ROS reduction at cytotoxic concentrations may be partly confounded by cell loss [41]. Therefore, 1 µm CLT was used as the representative effective concentration for subsequent in vitro validation and as a reference for hydrogel loading design (Figure 2h–j and Figure S1).

### 2.2 Preparation and Characterization of Hydrogels

The clinical translation of CLT is limited by its poor aqueous solubility and potential systemic toxicity. To improve local retention and enable sustained release, we developed an injectable hydrogel system for localized CLT delivery. The FGPC hydrogel was prepared through a micelle‐assisted loading and photo‐crosslinking process (Figure 3a). First, F127DA self‐assembled into micelles in aqueous solution, encapsulating hydrophobic CLT within their hydrophobic cores. These micelles, with diameters of ∼10–20 nm, are formed through hydrophobic interactions of PPO segments and effectively solubilize poorly water‐soluble compounds [42]. The initial CLT loading concentration in the FGPC pre–gel solution was 2 µm according to the formulation shown in Table S1, which was designed to approximate an effective released CLT concentration near 1 µm based on the subsequent release profile. GelMA was then introduced as a biocompatible network component, and PEDOT:PSS was incorporated to provide electrical conductivity. Finally, the pre–gel solution was photo‐crosslinked under 405 nm blue light irradiation to form the FGPC hydrogel.

**FIGURE 3:**
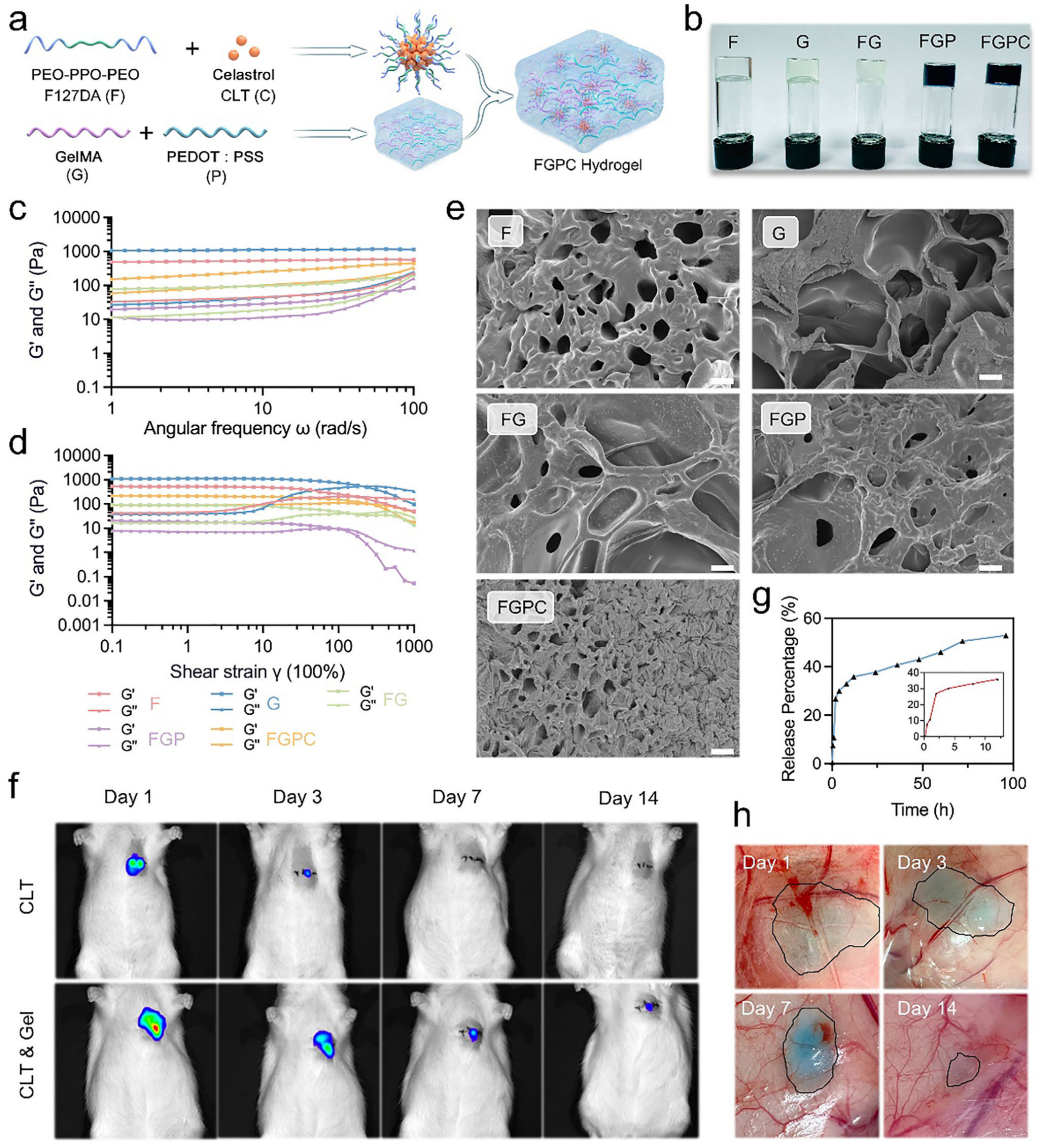
Preparation and characterization of hydrogels. (a) Schematic illustration of FGPC hydrogel fabrication. Celastrol was incorporated into F127DA micelles, followed by integration with GelMA and PEDOT:PSS to form the photo‐crosslinkable conductive hydrogel network. (b) Gross appearance of different hydrogel formulations after photo‐crosslinking. F, F127DA; G, GelMA; FG, F127DA/GelMA; FGP, F127DA/GelMA/PEDOT:PSS; FGPC, F127DA/GelMA/PEDOT:PSS/celastrol. (c) The storage modulus (*G*′) and loss modulus (*G*″) of the hydrogels under varying angular frequency. (d) The storage modulus (*G*′) and loss modulus (*G*″) of the hydrogels under varying shear strain (γ). (e) Representative SEM images of different hydrogels. Scale bars: 20 µm. (f) In vivo fluorescence imaging of free Cy5‐CLT and FGPC‐delivered Cy5‐CLT over 14 days. (g) In vitro cumulative release profile of Cy5‐CLT from FGPC hydrogel. (h) In vivo degradation of FGPC hydrogel in a subcutaneous implantation model.

The CLT‐loaded F127DA mixture remained homogeneous and stable after mixing and storage, supporting the role of F127DA micelles in improving CLT dispersion (Figure S2a). After PEDOT:PSS incorporation, the pre–gel solution showed a characteristic blue color. Upon 405 nm blue light irradiation, all formulations rapidly formed free‐standing hydrogels, as confirmed by vial inversion tests (Figure 3b). These results suggest that CLT loading and PEDOT:PSS incorporation did not interfere with hydrogel formation.

Photopolymerization produced a hydrogel with a crosslinked colloidal architecture, where covalent bridges between micelles provided structural integrity and hydrophobic interactions within the micellar network served as energy dissipation units, enabling flexible mechanical properties [32]. Rheological analysis was performed to evaluate the mechanical properties of different hydrogel formulations, including F127DA (F), GelMA (G), F127DA/GelMA (FG), F127DA/GelMA/PEDOT:PSS (FGP), and F127DA/GelMA/PEDOT:PSS/CLT (FGPC). The storage modulus (*G*′) remained higher than the loss modulus (*G*″) over the tested frequency range, indicating the formation of stable hydrogel networks (Figure 3c). Strain sweep analysis further confirmed the viscoelastic behavior of the hydrogels (Figure 3d). The incorporation of PEDOT:PSS and CLT did not markedly disrupt hydrogel gelation or mechanical stability. SEM imaging showed that all hydrogels exhibited porous microstructures, while PEDOT:PSS‐ and CLT‐containing hydrogels displayed relatively rougher internal surfaces (Figure 3e). These mechanical features may facilitate hydrogel adaptation to the dynamic myocardial surface. In addition, the electrical conductivity of the hydrogels was further quantified using a four‐point probe method. Incorporation of PEDOT:PSS significantly increased hydrogel conductivity, whereas CLT loading did not markedly alter the conductivity of the PEDOT:PSS‐containing matrix (Figure S5). These results confirm that PEDOT:PSS endowed the hydrogel with electrical conductivity, which may provide an electroactive microenvironment to support myocardial electrical coupling during repair.

To visualize local drug retention, Cy5‐labeled CLT was either locally applied in free form or encapsulated within FGPC hydrogel in the rat MIRI model. In vivo fluorescence imaging showed that the fluorescence signal in the free Cy5‐CLT group rapidly decreased, whereas FGPC‐delivered Cy5‐CLT remained detectable at the local application region over 14 days (Figure 3f). These results suggest that hydrogel encapsulation improved local retention of CLT. The in vitro release behavior of Cy5‐CLT from FGPC was then evaluated in PBS containing 0.1% Tween‐80. The release profile showed a biphasic pattern, with an initial release during the first 2 h followed by a slower sustained‐release phase, reaching approximately 52% cumulative release over 72 h (Figure 3g). Cy5‐CLT release was quantified using a fluorescence standard curve (Figure S2b), and representative release images further showed gradual diffusion of Cy5‐CLT from the hydrogel into the release medium (Figure S2c).

To further analyze the release mechanism, classical kinetic models were applied (Table S2). Among them, the Higuchi model showed the highest correlation coefficient (R^2^ = 0.8206), suggesting that diffusion played an important role in Cy5‐CLT release from FGPC hydrogel [43]. However, the release was not purely diffusion‐controlled. The Korsmeyer–Peppas fitting exponent (*n* = 0.5311), together with the swelling and degradation profiles, indicated that water uptake, polymer network relaxation, and progressive matrix degradation also participated in the sustained‐release process [44, 45]. Specifically, the swelling profile showed rapid early water uptake followed by gradual equilibration (Figure S4), whereas the degradation profile showed that the hydrogel network remained relatively intact during the early stage and gradually degraded over time (Figure S3). Therefore, Cy5‐CLT release from FGPC can be interpreted as a diffusion‐dominant but matrix‐assisted process, especially during the later sustained‐release phase.

Finally, in vivo hydrogel degradation was evaluated using a subcutaneous implantation model. FGPC gradually degraded after implantation and was nearly absorbed by day 14 (Figure 3h). Taken together, these results demonstrate that FGPC hydrogel provides stable gel formation, electrical conductivity, local drug retention, sustained Cy5‐CLT release, and gradual biodegradation.

### 2.3 FGPC Alters CLT Biodistribution and Prolongs Cardiac Retention

To further characterize the in vivo exposure pattern of CLT delivered by FGPC, liquid chromatography–tandem mass spectrometry (LC–MS/MS)‐based pharmacokinetic and biodistribution analyses were performed, and the experimental design is summarized in Figure S7a. Free CLT administered intraperitoneally was used as the systemic delivery control, whereas CLT‐loaded FGPC was locally applied to the epicardial surface after reperfusion. After intraperitoneal administration, CLT was readily detectable in plasma within 24 h, with an early peak followed by a progressive decline (Figure S7b). In contrast, CLT remained undetectable in plasma after local FGPC delivery over the monitored time window (Figure S7c). Biodistribution analysis showed that free CLT was mainly distributed in the liver and kidney, with lower levels detected in the spleen and lung, whereas sustained cardiac retention was not observed at the examined time points (Figure S7d). By contrast, FGPC‐delivered CLT was predominantly detected in the heart and remained measurable from day 1 to day 14, while showing minimal accumulation in the liver, spleen, lung, and kidney (Figure S7e).

These results indicate that FGPC altered the biodistribution profile of CLT by prolonging cardiac retention and reducing systemic exposure. The absence of detectable CLT in the heart after intraperitoneal administration should not be interpreted as a complete lack of myocardial access, but rather as limited cardiac retention under the current sampling schedule. Although these LC–MS/MS data support prolonged cardiac retention after FGPC delivery, they do not fully define the myocardial microregional concentration–time profile of released CLT.

### 2.4 In Vitro Biocompatibility, Conductive Function, and Antioxidant Activity of Hydrogels

Maintaining material biocompatibility is critical for clinical translation [46]. We therefore evaluated the cytocompatibility and functional effects of different hydrogels in vitro. Live/dead staining showed that most AC16 cells cultured on different hydrogels remained viable over 5 days, with only sporadic dead cells observed (Figure 4a). Consistently, CCK‐8 analysis showed increased metabolic activity from day 1 to day 5, indicating that the hydrogel formulations did not inhibit AC16 cell proliferation (Figure 4b). Overall, these results suggest that the hydrogels exhibited good cytocompatibility.

**FIGURE 4:**
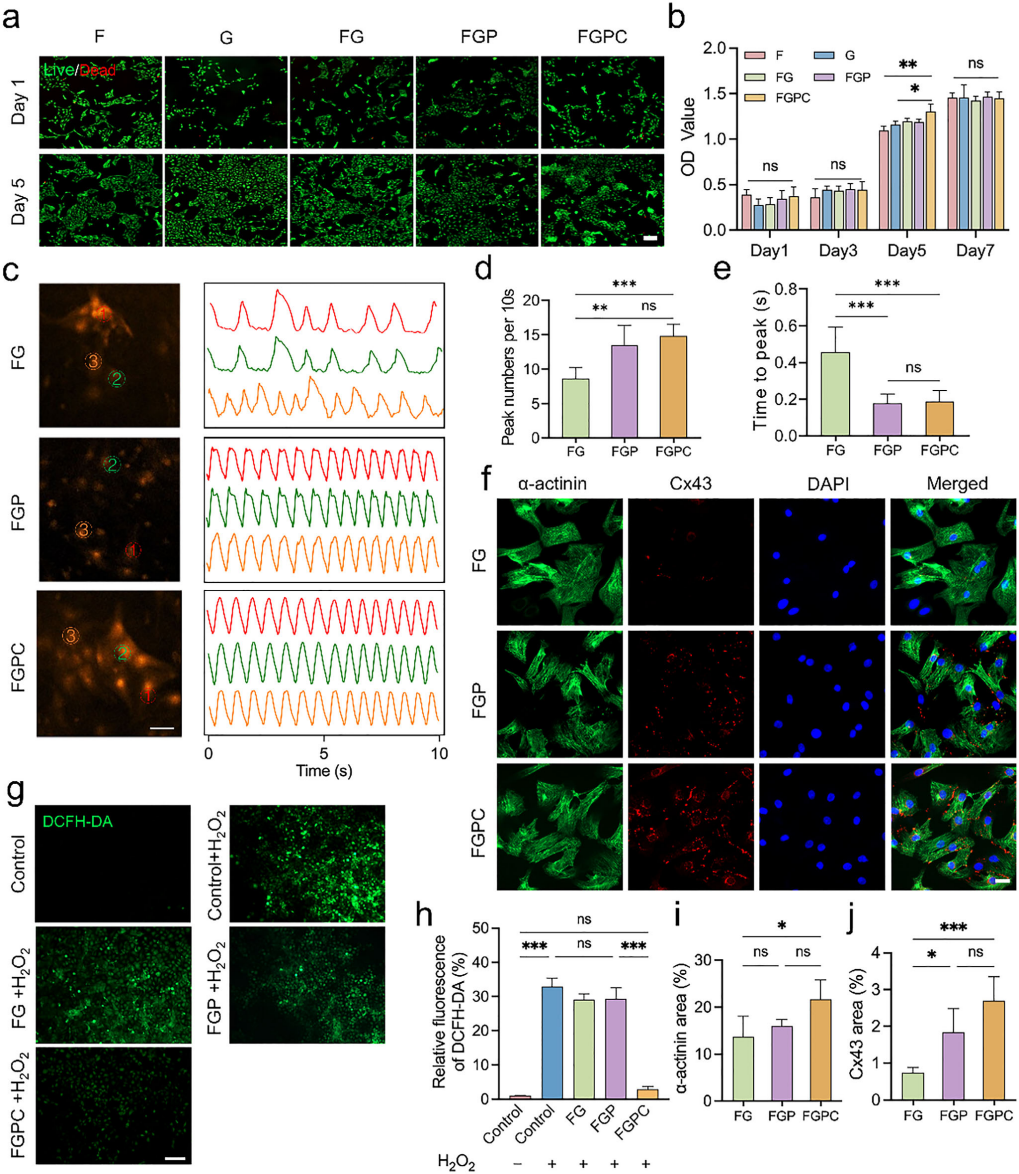
In vitro biocompatibility, antioxidant, and electroconductive properties of different hydrogels. (a) Representative Live/dead staining of AC16 cells cultured on different hydrogels at days 1 and 5. Green: live cells; red: dead cells. Scale bar: 200 µm. (b) CCK‐8 analysis of AC16 cell proliferation on different hydrogels. (c) Representative Ca^2^^+^ transient images and traces of neonatal rat ventricular cardiomyocytes (NRVMs) cultured on FG, FGP, and FGPC hydrogels for 7 days (*n* = 3). (Left) Representative images of calcium transients. (Right) Quantitative analysis of the calcium transient frequency. Scale bar: 50 µm. (d, e) Quantification of Ca^2^^+^ transient peak number per 10 s (d) and time to peak (e) (*n* = 6). (f) Representative immunofluorescence images of α‐actinin and Cx43 in cardiomyocytes cultured on different hydrogels. Green, α‐actinin; red, Cx43; blue, DAPI. Scale bar, 20 µm. (g) Representative DCFH‐DA fluorescence images of AC16 cells cultured on different hydrogels under H_2_O_2_‐induced oxidative stress. Scale bar, 200 µm. (h) Quantification of intracellular ROS fluorescence intensity (*n* = 4). (i, j) Quantification of α‐actinin‐positive area (i) and Cx43‐positive area (j) (*n* = 6). Data are presented as mean ± SD. Statistical significance was determined by one‐way ANOVA followed by Tukey's post hoc test. (^*^ *p* < 0.05, ^**^ *p* < 0.01, ^***^ *p* < 0.001; ns, not significant.).

Conductive hydrogels have been reported to support cardiomyocyte function by enhancing intercellular electrical communication and electrophysiological synchronization [47, 48]. In this study, we examined calcium transient activity and structural organization in neonatal rat ventricular cardiomyocytes (NRVMs) cultured on different hydrogels. NRVMs cultured on the non‐conductive FG hydrogel showed relatively weak and less synchronized Ca^2^^+^ transient activity, whereas cells cultured on PEDOT:PSS‐containing FGP and FGPC hydrogels exhibited more synchronized Ca^2^^+^ transients (Figure 4c). Quantitative analysis showed that FGP and FGPC increased the number of Ca^2^^+^ transient peaks and shortened the time to peak compared with FG (Figure 4d,e). Because FGP and FGPC showed comparable effects, these improvements were mainly associated with the conductive PEDOT:PSS‐containing matrix rather than CLT loading. Immunofluorescence staining further showed improved α‐actinin organization and increased Cx43 expression in cardiomyocytes cultured on conductive hydrogels (Figure 4f,i,j), suggesting enhanced structural organization and gap‐junction formation.

Moreover, myocardial infarction induces excessive reactive oxygen species (ROS) in the infarcted zone, causing oxidative damage [49]. Celastrol (CLT) has shown promise in ischemia/reperfusion contexts, such as by inhibiting HMGB1 binding to inflammatory receptors under hypoxic conditions. It also exhibits neuroprotective and anti‐inflammatory effects in neural I/R injury [50], along with anti‐inflammatory and anti‐apoptotic roles in diet‐induced myocardial injury [39]. To evaluate the ROS‐scavenging capacity of our hydrogels, we measured intracellular ROS using DCFH‐DA staining. AC16 cells cultured on hydrogels without CLT showed strong DCFH‐DA fluorescence, indicating increased intracellular ROS levels. In contrast, cells cultured on CLT‐loaded FGPC hydrogel exhibited reduced DCFH‐DA fluorescence intensity (Figure 4g,h). These results indicate that CLT loading endowed FGPC with enhanced ROS‐scavenging capacity in vitro, supporting its potential to modulate the oxidative microenvironment during myocardial ischemia–reperfusion injury.

### 2.5 FGPC Attenuates Acute Oxidative Stress and Inflammatory Activation In Vivo

Based on its in vitro cytocompatibility, ROS‐scavenging capacity, and conductive support for cardiomyocyte function, FGPC was further evaluated in a rat myocardial ischemia–reperfusion injury model. Myocardial ischemia was induced by transient ligation of the left anterior descending coronary artery for 30 min, followed by reperfusion. Immediately after reperfusion, the pre‐gel solution was locally applied onto the epicardial surface of the ischemic region and photo‐crosslinked in situ using 405 nm blue light. Successful ischemia was indicated by pale discoloration of the left ventricular wall, reduced wall motion, and ST‐segment elevation on electrocardiogram(ECG), followed by partial ST‐segment recovery after reperfusion (Figure S9a). The experimental timeline included acute assessment at 24 h and chronic evaluation up to 4 weeks after surgery, including echocardiography, electrophysiological examination, and histological analysis (Figure 5a).

**FIGURE 5:**
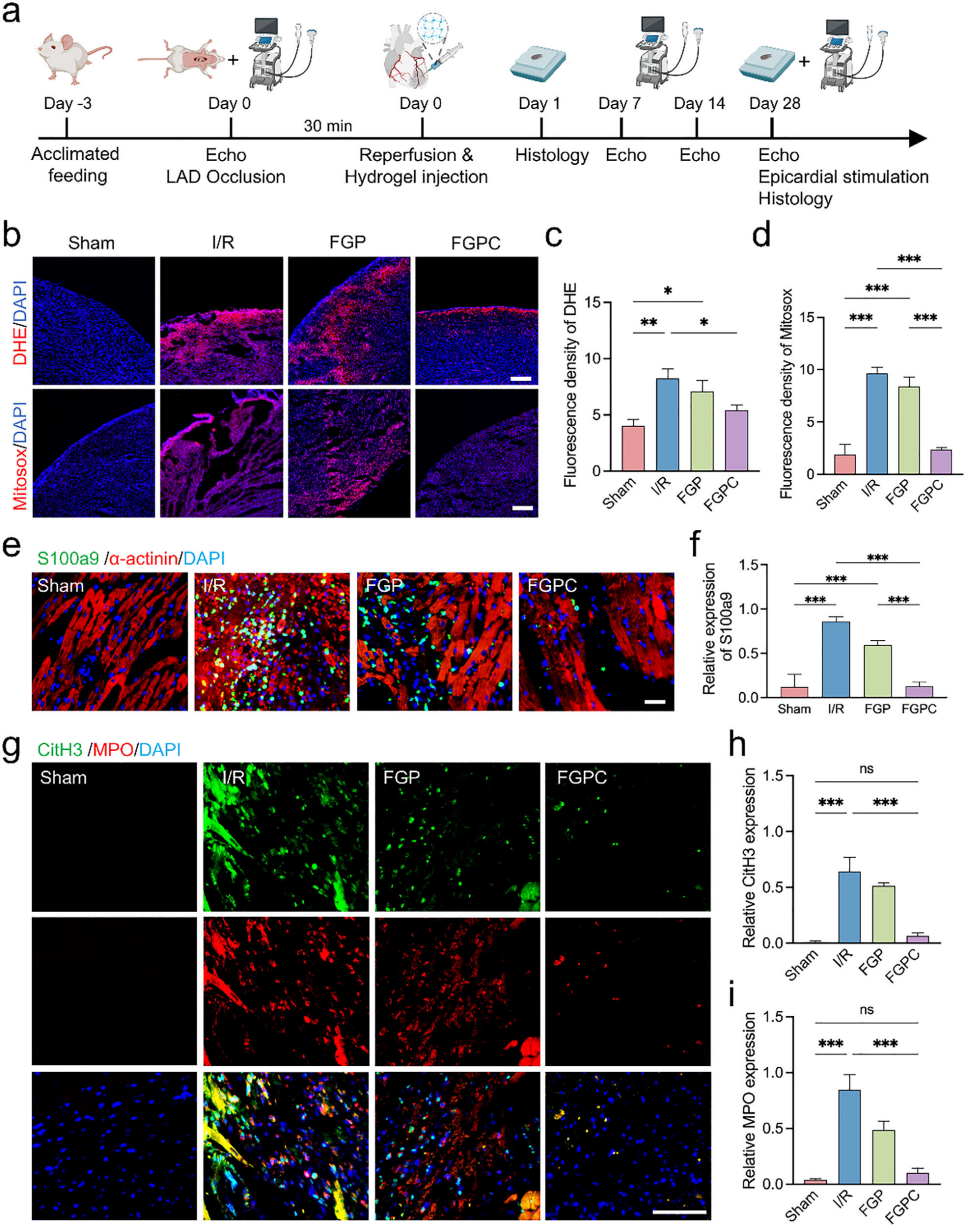
FGPC reduces acute oxidative stress and inflammation in vivo. (a) Experimental timeline of MI/R induction, hydrogel treatment, cardiac functional assessment, electrophysiological examination, and tissue collection. (b) Representative fluorescence images of DHE and MitoSOX staining in heart sections at 24 h after reperfusion. Red, DHE or MitoSOX; blue, DAPI. Scale bars, 200 µm. (c, d) Quantification of DHE (c) and MitoSOX (d) fluorescence intensity. (e) Representative immunofluorescence images of S100a9 in heart sections at 24 h after reperfusion. Green, S100a9; red, α‐actinin; blue, DAPI. Scale bar, 50 µm. (f) Quantification of S100a9 fluorescence intensity. (g) Representative immunofluorescence images of CitH3 and MPO in heart sections at 24 h after reperfusion. Green, CitH3; red, MPO; blue, DAPI. Scale bar, 50 µm. (h, i) Quantification of CitH3 (h) and MPO (i) fluorescence intensity. Data are presented as mean ± SD (*n* = 5). Statistical significance was determined by one‐way ANOVA followed by Tukey's post hoc test. (^*^ *p* < 0.05, ^**^ *p* < 0.01, ^***^ *p* < 0.001; ns, not significant.).

Excessive ROS generation is a key early event in MIRI and contributes to subsequent inflammatory amplification. At 24 h after reperfusion, dihydroethidium (DHE) and MitoSOX staining showed markedly increased fluorescence signals in the I/R group compared with the Sham group, indicating elevated total and mitochondrial ROS levels in the injured myocardium (Figure 5b–d). In contrast, FGPC‐treated hearts exhibited significantly reduced DHE and MitoSOX fluorescence intensity, suggesting that local CLT delivery through FGPC attenuated oxidative stress during the acute phase of reperfusion injury. Early neutrophil infiltration is a major contributor to reperfusion‐associated injury because activated neutrophils release proinflammatory cytokines, chemokines, proteases, and NETs, thereby exacerbating cardiomyocyte death and tissue damage [13]. Immunofluorescence staining showed increased S100a9 expression in the I/R group, whereas FGPC treatment markedly reduced S100a9 fluorescence intensity (Figure 5e,f). Similarly, the NETosis‐related markers citrullinated histone H3 (CitH3) and myeloperoxidase (MPO) were elevated after I/R injury but were significantly decreased in the FGPC group (Figure 5g–i). These findings indicate that FGPC suppressed early inflammatory activation and NET formation after reperfusion.

To further compare FGPC with free‐drug administration, acute myocardial injury was evaluated by Evans blue/2,3,5‐triphenyltetrazolium chloride (TTC) staining and plasma CK‐MB/cTnI measurements at 24 h after reperfusion. FGPC reduced infarct area/left ventricular area (INF/LV) and plasma injury‐marker levels compared with free CLT under the present experimental conditions, supporting the advantage of hydrogel‐mediated local delivery for acute myocardial protection (Figure S8). Taken together, these results suggest that FGPC mitigates acute myocardial injury after reperfusion by reducing ROS accumulation, suppressing S100a9‐associated inflammatory activation, and inhibiting NET formation.

### 2.6 RNA Sequencing Reveals Inflammatory Pathway Suppression by FGPC

To investigate the transcriptomic changes associated with FGPC treatment during the acute phase of MIRI, RNA sequencing (RNA‐seq) was performed on infarct border‐zone tissues collected at 24 h after reperfusion, with *n* = 3 biologically independent samples per group. Principal component analysis showed separation between the I/R and FGPC groups, suggesting distinct transcriptional profiles after treatment (Figure 6a). Compared with the I/R group, FGPC treatment resulted in 772 differentially expressed genes, including 270 upregulated and 502 downregulated genes (Figure 6b).

**FIGURE 6:**
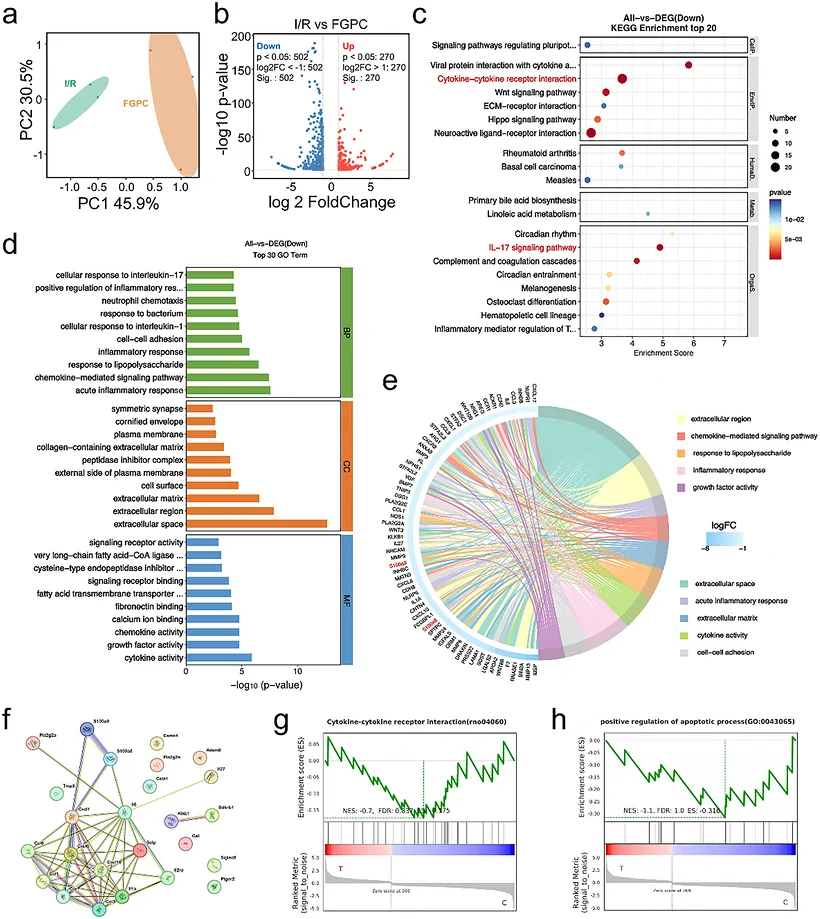
RNA‐seq reveals inflammatory pathway suppression by FGPC (*n* = 3). (a) Principal component analysis (PCA) of RNA‐seq samples from the I/R and FGPC groups. (b) Volcano plot identifying differentially expressed genes (DEGs) in myocardial tissue between the FGPC and I/R groups (DEGs were defined as FDR < 0.05 and |log_2_FC| ≥ 1). (c) Kyoto Encyclopedia of Genes and Genomes (KEGG) pathway enrichment analysis of downregulated differentially expressed genes in the FGPC group compared with the I/R group. (d) Gene ontology (GO) enrichment analysis of downregulated differentially expressed genes, including biological process, cellular component, and molecular function terms. (e) Chord diagram showing representative downregulated genes and their associated enriched GO terms. The color gradient indicates relative log_2_ fold change. (f) STRING protein–protein interaction (PPI) network of representative downregulated inflammation‐related genes. (g, h) Gene set enrichment analysis (GSEA) plots of cytokine–cytokine receptor interaction (g) and positive regulation of the apoptotic process (h).

Functional enrichment analysis of the downregulated genes showed that FGPC treatment was mainly associated with reduced inflammatory and immune‐related responses. Gene ontology (GO) biological process terms included cellular response to interleukin‐1, neutrophil chemotaxis, and inflammatory response, while enriched molecular function terms included signaling receptor activity, chemokine activity, and cytokine activity (Figure 6d,e). Several inflammation‐related genes, including *Il6*, *Ccl3*, *Cxcl1*, *Cxcl10*, *S100a8*, and *S100a9*, were downregulated in the FGPC group, suggesting attenuation of cytokine‐ and chemokine‐mediated inflammatory activation after reperfusion. Kyoto Encyclopedia of Genes and Genomes (KEGG) pathway analysis further supported this inflammatory regulatory pattern. Downregulated genes were enriched in pathways related to cytokine–cytokine receptor interaction, IL‐17 signaling pathway, complement and coagulation cascades, and other immune‐related processes (Figure 6c). These results are consistent with the early reduction in S100a9 expression and NETosis‐related markers observed by immunofluorescence staining, suggesting that FGPC attenuates inflammatory amplification during the acute phase of MIRI.

To further explore inflammation‐associated gene interactions, a protein‐protein interaction (PPI) network was constructed using representative downregulated genes. The network highlighted *S100a8* and *S100a9* as central inflammation‐related nodes connected with chemokines and cytokines such as *Cxcl1*, *Cxcl2*, and *Il1b* (Figure 6f). Given that S100a8/a9 can function as a damage‐associated molecular pattern and participate in neutrophil recruitment and inflammatory signaling during tissue injury [51, 52, 53], its downregulation may contribute to the reduced inflammatory response observed after FGPC treatment. Gene set enrichment analysis (GSEA) provided additional evidence of pathway‐specific regulation. The cytokine–cytokine receptor interaction pathway and positive regulation of the apoptotic process were reduced in the FGPC group compared with the I/R group (Figure 6g,h), indicating that FGPC treatment was associated with coordinated suppression of inflammatory signaling and apoptosis‐related transcriptional programs.

To corroborate the transcriptomic findings at the mRNA level, representative inflammation‐related genes were selected for qPCR validation using independent myocardial samples. Consistent with the sequencing results, *S100a8*, *S100a9*, *Il6*, and *Cxcl1* were increased after I/R injury and were reduced after FGPC treatment (Figure S6). Together, these transcriptomic and qPCR data support that FGPC suppresses early inflammatory activation in the infarct border zone after reperfusion.

### 2.7 FGPC Attenuates Fibrosis and Improves Cardiac Function In Vivo

At 4 weeks after I/R surgery, Masson's trichrome staining was performed to evaluate myocardial fibrosis and scar formation. Compared with the Sham group, the I/R group showed extensive collagen deposition and an increased fibrotic area in the left ventricular infarct region (Figure 7a,b). Fibrotic deposition remained evident in the FGP group and was comparable to that observed in the I/R group. In contrast, FGPC treatment substantially reduced collagen deposition and fibrotic area, suggesting improved structural preservation after reperfusion injury.

**FIGURE 7:**
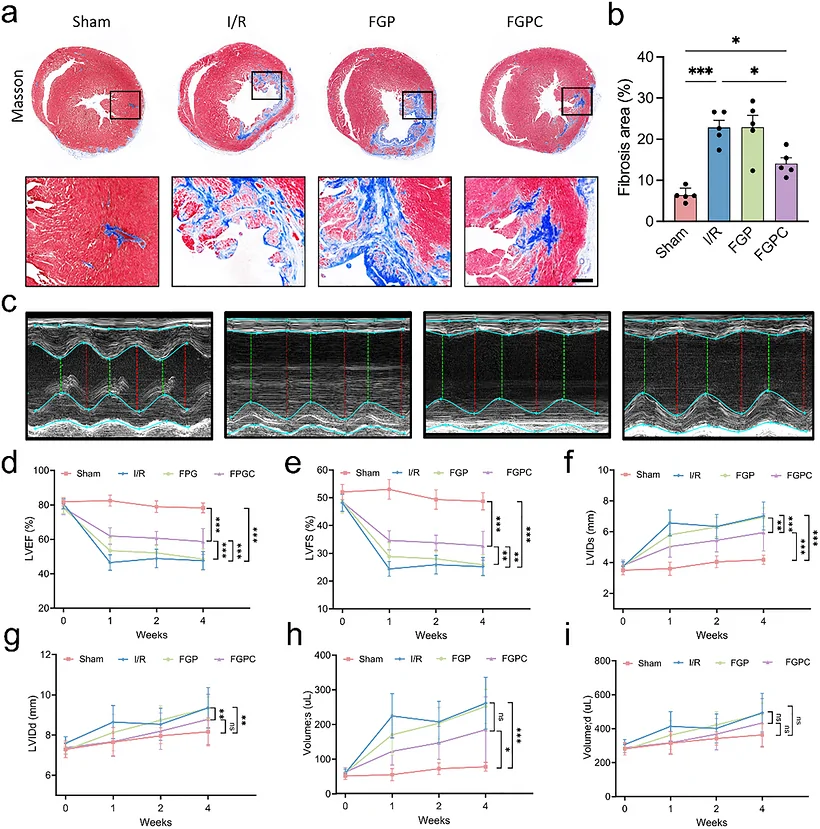
FGPC attenuates myocardial fibrosis and improves cardiac function after myocardial ischemia–reperfusion injury. (a) Representative Masson's trichrome staining images of myocardial sections from the Sham, I/R, FGP, and FGPC groups at 4 weeks after treatment. The lower panels show higher‐magnification views of the boxed regions in the corresponding upper panels. Collagen fibers are stained blue. Scale bars: 500 µm. (b) Quantitative analysis of fibrotic area in each group. (c) Representative M‐mode echocardiographic images of each group at 4 weeks after surgery. The green and red dashed lines indicate the left ventricular internal diameters at end‐diastole and end‐systole, respectively. (d–i) Longitudinal echocardiographic analysis of left ventricular function from baseline to 4 weeks after surgery, including LVEF (d), LVFS (e), LVIDs (f), LVIDd (g), LV systolic volume (Volume;s) (h), and LV diastolic volume (Volume;d) (i). Data are presented as mean ± SD (*n* = 8). Statistical significance was determined by one‐way ANOVA followed by Tukey's post hoc test for comparisons at 4 weeks. (^*^ *p* < 0.05, ^**^ *p* < 0.01, ^***^ *p* < 0.001; ns, not significant).

Cardiac function was further assessed by serial echocardiography from baseline to 4 weeks after surgery. Representative M‐mode echocardiographic images at 4 weeks showed impaired left ventricular wall motion and chamber dilation in the I/R group, whereas these changes were alleviated in the FGPC group (Figure 7c). Quantitative longitudinal analysis showed that, after I/R injury, the untreated I/R group exhibited reduced left ventricular ejection fraction (LVEF) and fractional shortening (LVFS), together with progressive ventricular dilation. In contrast, FGPC treatment improved LVEF and LVFS, with the most evident differences observed at 4 weeks after surgery (Figure 7d,e). FGPC also reduced left ventricular internal diameter at systole (LVIDs) and diastole (LVIDd), as well as systolic and diastolic LV volumes, indicating attenuation of ventricular dilation and adverse remodeling (Figure 7f–i). Together, these results show that FGPC reduced myocardial fibrosis and improved cardiac functional recovery after I/R injury.

### 2.8 FGPC Promotes Electrophysiological and Vascular Repair In Vivo

Synchronous cardiomyocyte contraction critically depends on the structural protein α‐actinin, which is essential for sarcomere integrity and muscle contraction [54]. Cardiomyocyte structural organization and gap‐junction remodeling were further evaluated at 4 weeks after I/R surgery. Immunofluorescence staining showed reduced α‐actinin organization and Cx43 expression in the I/R group, indicating impaired myocardial structural integrity and electrical coupling after reperfusion injury (Figure 8a–c). FGPC treatment increased the α‐actinin‐positive area and restored Cx43 expression in the infarct border zone compared with the I/R group. These results suggest that FGPC contributed to improved cardiomyocyte structural organization and gap‐junction remodeling during chronic repair.

**FIGURE 8:**
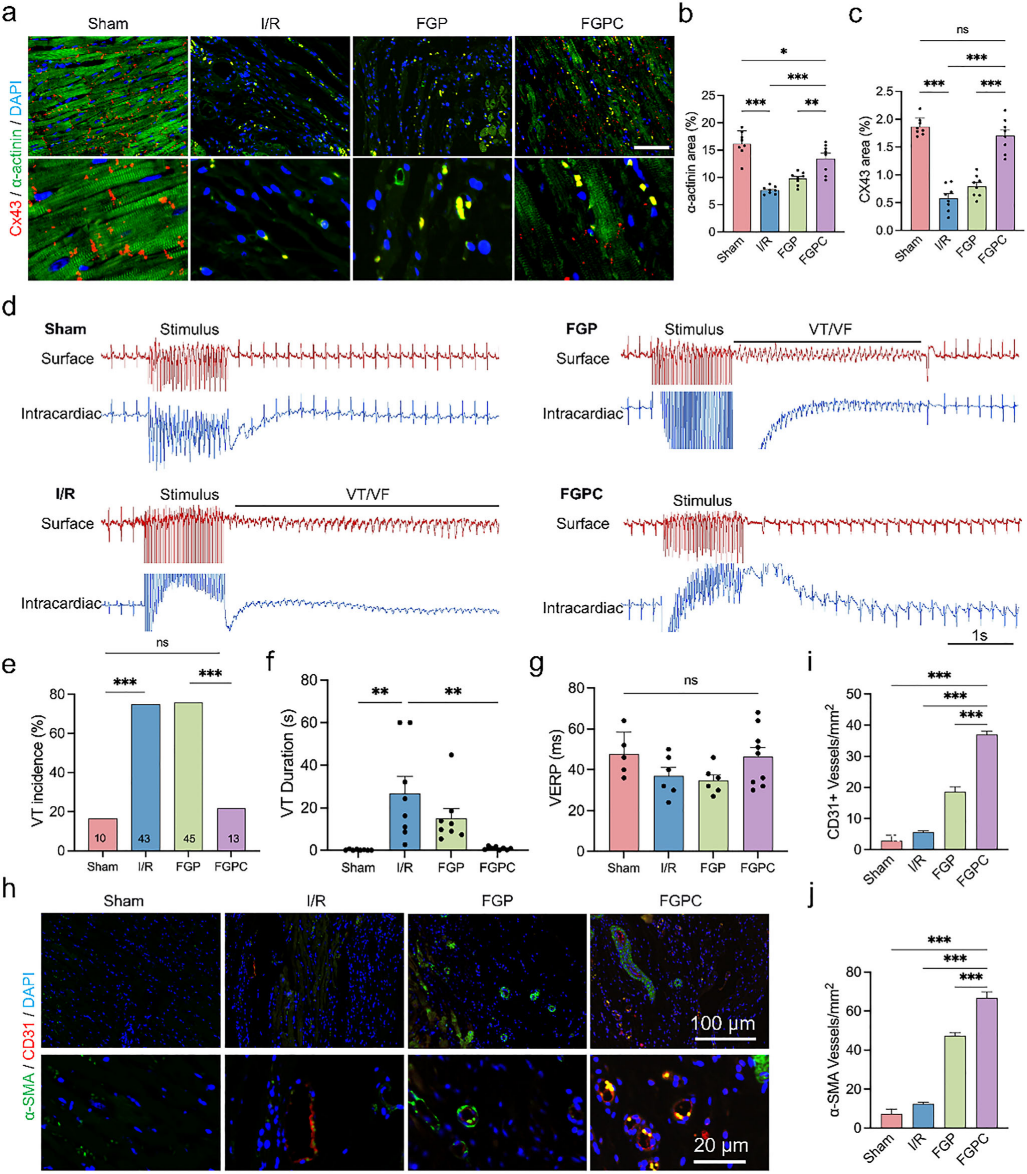
FGPC improves electrical stability and neovascularization in vivo. (a) Representative immunofluorescence images of α‐actinin and Cx43 in the infarct border zone at 4 weeks after surgery. Green, α‐actinin; red, Cx43; blue, DAPI. Scale bars: 50 and 10 µm for magnified images. (b, c) Quantification of α‐actinin‐positive area (b) and Cx43‐positive area (c). (d) Representative surface ECG and intracardiac electrograms during programmed electrical stimulation. VT/VF, ventricular tachycardia/ventricular fibrillation. Scale bar, 1 s. (e–g) Quantification of VT/VF incidence (e), VT duration (f), and ventricular effective refractory period (VERP) (g). VT: ventricular tachycardia; VF: ventricular fibrillation. VERP: Ventricular Effective Refractory Period. (h) Representative immunofluorescence images of CD31 and α‐SMA in cardiac sections at 4 weeks after surgery. Red, CD31; green, α‐SMA; blue, DAPI. Scale bars: 100 and 20 µm for magnified images. (i, j) Quantification of CD31‐positive vessel density (i) and α‐SMA‐positive vessel density (j). Except for the data of VT/VF incidence, the other data are presented as mean ± SD (*n* = 8). Statistical significance was determined by one‐way ANOVA followed by Tukey's post hoc test for continuous variables. VT/VF incidence was analyzed using Fisher's exact test or chi‐square test. (^*^ *p* < 0.05, ^**^ *p* < 0.01, ^***^ *p* < 0.001; ns, not significant.).

The effect of FGPC on ventricular arrhythmia susceptibility was then assessed at 4 weeks after treatment using programmed electrical stimulation (PES). The I/R group showed a high incidence of inducible ventricular arrhythmias during S1–S2 stimulation, whereas FGPC‐treated rats exhibited fewer sustained VT/VF episodes (Figure 8d,e and Figure S10). Although the difference in ventricular effective refractory period (VERP) did not reach statistical significance, FGPC treatment reduced VT duration and lowered arrhythmia inducibility compared with the I/R group (Figure 8f,g). These findings suggest that FGPC improved post‐I/R electrical stability. This effect may be related to the combined contribution of the PEDOT:PSS‐containing conductive matrix and CLT‐mediated attenuation of early oxidative and inflammatory injury, which together may help preserve a more favorable substrate for electrical remodeling.

Revascularization of the injured myocardium is crucial for functional recovery after infarction. Enhancing angiogenesis represents an effective strategy for restoring oxygen and nutrient supply to affected areas [55, 56]. Neovascularization in the infarct border zone was evaluated by immunofluorescence staining for endothelial cells (CD31) and vascular smooth muscle cells (α‐SMA) at 4 weeks after surgery. Compared with the I/R group, FGPC treatment increased CD31‐positive and α‐SMA‐positive vessel density, indicating enhanced angiogenesis and vessel maturation during myocardial repair (Figure 8h–j). This pro‐angiogenic effect may be related to the improved local repair microenvironment created by FGPC after reperfusion injury. Together, the increased CD31‐ and α‐SMA‐positive vessel density suggests that FGPC promoted vascular repair in the injured myocardium.

The comparison between FGP and FGPC helps clarify the relative contribution of CLT loading to the long‐term therapeutic outcome. Both FGP and FGPC contained the PEDOT:PSS‐based conductive hydrogel matrix, whereas FGPC additionally provided sustained local CLT delivery. In vivo, compared with the non‐drug FGP hydrogel, FGPC more effectively reduced acute ROS accumulation, S100a9 expression, NETosis‐related markers, and inflammatory pathway activation [52]. Because early oxidative stress and inflammatory amplification after reperfusion can promote subsequent fibrotic remodeling, ventricular dilation, gap‐junction disturbance, and arrhythmogenic substrate formation, the additional reduction in fibrosis and the greater improvement in LVEF/LVFS observed in the FGPC group are likely related to CLT‐mediated attenuation of early oxidative and inflammatory injury [57, 58]. Notably, although both FGP and FGPC contained PEDOT:PSS, FGPC showed a more evident reduction in inducible ventricular arrhythmia susceptibility and VT/VF burden, suggesting that conductive support alone may be insufficient to fully prevent post‐I/R electrical instability when early oxidative and inflammatory injury persists. Taken together, the additional improvements observed in the FGPC group are likely related to early CLT‐mediated suppression of oxidative and inflammatory injury, which may limit the development of a fibrotic and arrhythmogenic substrate during chronic repair. The PEDOT:PSS‐containing matrix may further support this process by providing a conductive microenvironment favorable for electrical and structural remodeling.

These findings also place FGPC within the broader context of local delivery strategies for MIRI. Compared with systemic free‐drug administration, hydrogel‐mediated local delivery can improve myocardial drug retention and reduce systemic exposure, which is particularly relevant for CLT because of its poor aqueous solubility and potential systemic toxicity [59]. Consistently, FGPC prolonged cardiac CLT retention, reduced plasma and off‐target organ exposure, and provided stronger acute myocardial protection than free CLT administration. In addition, unlike conventional non‐conductive drug depots, FGPC incorporates PEDOT:PSS‐mediated conductive support to help modulate the impaired electrical microenvironment after I/R injury [60]. Thus, FGPC represents an integrated local delivery platform that combines sustained CLT exposure with conductive microenvironment support.

### 2.9 Preliminary Biosafety Evaluation of FGPC Hydrogel

Given the potential systemic toxicity of CLT, the preliminary biosafety of FGPC treatment was evaluated at 4 weeks after surgery. H&E staining of major organs, including the heart, liver, kidney, and testis, showed no obvious histological abnormalities in the FGPC group (Figure S11a). Serum biochemical analysis showed no significant changes in ALT, AST, CREA, or BUN levels among groups, suggesting that liver and kidney function were not markedly affected under the present experimental conditions (Figure S11b–e). In addition, the percentage of circulating neutrophils among total white blood cells showed no significant increase after FGPC treatment (Figure S11f). These results suggest that local FGPC application did not induce obvious systemic toxicity during the observation period.

## 3 Conclusion

In this study, we developed a celastrol‐loaded conductive hydrogel, FGPC, for localized drug delivery and microenvironment modulation after myocardial ischemia–reperfusion injury. Through phenotype‐guided antioxidant screening, CLT was selected as a bioactive cargo and incorporated into a GelMA/F127DA/PEDOT:PSS hydrogel system. The resulting FGPC hydrogel enabled local CLT retention, sustained release, and conductive microenvironment support. In vitro, FGPC exhibited good cytocompatibility, reduced ROS accumulation under oxidative stress, and supported cardiomyocyte structural organization and Ca^2^^+^ transient propagation. In vivo, local FGPC application attenuated acute oxidative stress, S100a9‐associated inflammatory activation, and NET formation, while transcriptomic analysis further indicated suppression of inflammatory pathways. During the chronic repair phase, FGPC reduced myocardial fibrosis, improved cardiac function, promoted gap‐junction remodeling and neovascularization, and reduced inducible ventricular arrhythmia susceptibility without obvious systemic toxicity. Overall, this study highlights a locally retained conductive hydrogel platform that combines CLT‐mediated antioxidative and anti‐inflammatory effects with electrical microenvironment support, providing a potential strategy for myocardial repair after reperfusion.

## 4 Experimental Section

### 4.1 Drug Screening and Celastrol Validation

A 96‐well antioxidant compound library (Selleck, L6500‐01) was used to screen candidate antioxidants in AC16 cells under oxidative stress. Briefly, AC16 cells were seeded in 96‐well plates and pretreated with library compounds at 1 or 10 µm for 2 h, followed by exposure to 400 µm H_2_O_2_ for 4 h. Intracellular ROS levels were assessed using a DCFH‐DA probe, and compounds showing the strongest ROS reduction were selected for further evaluation. Celastrol (CLT) was subsequently tested over a concentration range of 0.001–10 µm to evaluate its antioxidant efficacy and cytocompatibility.

### 4.2 Synthesis of Hydrogel

F127DA powder was dissolved in deionized water precooled to 4°C, and GelMA lyophilizate was dissolved in deionized water at 50°C. For CLT‐loaded FGPC hydrogels, CLT was first mixed with the F127DA solution at 4°C in the dark to facilitate micellar encapsulation. After the F127DA and GelMA solutions reached room temperature, GelMA, PEDOT:PSS dispersion, and LAP photoinitiator solution were sequentially added and thoroughly mixed. The final FGPC pre–gel solution contained an initial CLT loading concentration of 2 µm, 10 wt.% F127DA, 7.5 wt.% GelMA, 0.1 wt.% PEDOT:PSS, and 0.25 wt.% lithium phenyl‐2,4,6‐trimethylbenzoylphosphinate (LAP) photoinitiator. The pre–gel solution was exposed to 405 nm blue light irradiation for 60 s to form the crosslinked hydrogel. Non‐drug hydrogels were prepared using the same procedure without CLT addition.

### 4.3 Hydrogel Characterization

The microstructure of the hydrogels was examined by scanning electron microscopy (SEM) after lyophilization. Rheological properties were measured using an oscillatory rheometer at 37°C. Storage modulus (*G*′) and loss modulus (*G*″) were recorded as a function of frequency (1–100 rad/s). Critical strain was determined using strain sweep tests, with the *G*′–*G*″ crossover point representing the limit of the linear viscoelastic region (strain range: 0.1%–1000%).

### 4.4 In Vitro Release of Cy5‐CLT From FGPC Hydrogel

Cy5‐CLT‐loaded FGPC hydrogels were immersed in 5 mL of release medium consisting of PBS containing 0.1% Tween‐80 and incubated at 37°C with gentle shaking. At predetermined time points, 500 µL of the release medium was collected and replaced with an equal volume of fresh prewarmed medium. The released Cy5‐CLT was quantified using a fluorescence microplate reader at an excitation wavelength of 646 nm and an emission wavelength of 662 nm. Cy5‐CLT concentrations were calculated from a standard curve generated under the same conditions. The cumulative release percentage was calculated after correction for medium replacement and expressed relative to the initial amount of Cy5‐CLT loaded in the hydrogel.

### 4.5 Pharmacokinetic and Biodistribution Analysis of Celastrol

Free celastrol was administered intraperitoneally as the systemic delivery control, whereas FGPC containing celastrol was locally applied to the epicardial surface after reperfusion. Blood samples were collected at predetermined time points within 24 h for plasma concentration analysis. Major organs, including the heart, liver, spleen, lung, and kidney, were harvested at 1, 3, 7, and 14 days after administration. Celastrol concentrations in plasma and tissue samples were quantified by LC–MS/MS. Three biologically independent samples were analyzed for each condition. The overall sampling strategy is illustrated in Figure S7a.

### 4.6 In Vitro Evaluation of Hydrogel Biocompatibility and Antioxidant Activity

AC16 cells were seeded onto hydrogel samples to evaluate cytocompatibility and antioxidant activity. Cell viability and proliferation were assessed using the CCK‐8 assay on days 1, 3, 5, and 7, and Live/Dead staining was performed using Calcein‐AM and propidium iodide. To evaluate antioxidant activity, AC16 cells cultured on hydrogels for 24 h were exposed to 400 µm H_2_O_2_ for 4 h, followed by DCFH‐DA staining to assess intracellular ROS levels. Fluorescence images were acquired using a fluorescence microscope.

### 4.7 In Vitro Assessment of Cardiomyocyte Structure and Calcium Transients

Neonatal rat cardiomyocytes were isolated from 1–3‐day‐old Sprague‐Dawley rats and seeded onto hydrogel samples. After 7 days of culture, cardiomyocyte structural organization and intercellular coupling were evaluated by immunofluorescence staining for α‐actinin and Cx43. For calcium transient analysis, cardiomyocytes were stained with Fluo‐4 AM and imaged under a fluorescence microscope to assess spontaneous Ca^2^^+^ transient activity.

### 4.8 Myocardial Ischemia–Reperfusion (MI/R) Rat Model and Hydrogel Treatment

Male Sprague‐Dawley rats weighing approximately 220 g were anesthetized with 2% isoflurane, intubated, and mechanically ventilated. After left thoracotomy, the left anterior descending coronary artery was ligated with a 6‐0 suture for 30 min to induce myocardial ischemia, followed by ligature release to initiate reperfusion. Successful ischemia was confirmed by blanching of the left ventricular anterior wall and ST‐segment elevation on ECG. Immediately after reperfusion, 200 µL of pre–gel solution was applied onto the epicardial surface of the ischemic region and crosslinked in situ using 405 nm blue light irradiation. Sham‐operated rats underwent the same surgical procedure without left anterior descending (LAD) ligation. Animals were randomly assigned to four groups: Sham (thoracotomy without LAD ligation, *n* = 8), I/R (MI/R without hydrogel treatment, *n* = 8), FGP (MI/R with non‐drug conductive hydrogel, *n* = 8), and FGPC (MI/R with CLT‐loaded conductive hydrogel, *n* = 8). All animal procedures were approved by the Animal Ethics Committee of Nanjing Medical University (IACUC‐2504050).

### 4.9 Echocardiographic Evaluation of Cardiac Function

Transthoracic echocardiography was performed preoperatively and at 1, 2, and 4 weeks after surgery using a Vevo 2100 imaging system. Rats were anesthetized with isoflurane during image acquisition. Two‐dimensional guided M‐mode images were obtained from the short‐axis view at the papillary muscle level. Left ventricular ejection fraction (LVEF), fractional shortening (LVFS), left ventricular internal diameter at systole (LVIDs), and left ventricular internal diameter at diastole (LVIDd) were measured to evaluate cardiac function.

### 4.10 RNA Sequencing and Bioinformatics Analysis

Myocardial tissues were collected from the infarct border zone at 24 h after reperfusion for RNA sequencing. Three biologically independent samples from each group were analyzed. RNA sequencing was performed on the Illumina NovaSeq platform by Shanghai Hongxu Biotechnology Co., Ltd. Differentially expressed genes were identified using edgeR, with an FDR < 0.05 and |log_2_FC| ≥ 1 considered significant. GO and KEGG enrichment analyses were performed using the clusterProfiler package, and pathways with adjusted *p* < 0.05 were considered significantly enriched.

### 4.11 Programmed Electrical Stimulation (PES)

Four weeks after hydrogel injection, arrhythmia inducibility was evaluated using a clinically standardized PES protocol. Under inhalation anesthesia, an octapolar catheter (CI'BER Mouse, NuMed) was advanced into the right ventricle via the right jugular vein under ECG guidance. Extrastimulus (S1–S2) and burst pacing (0.5–1 s) protocols were performed as previously described. Ventricular tachycardia (VT) was defined as ≥4 consecutive ventricular beats with atrioventricular dissociation.

### 4.12 Immunofluorescence Analysis of Acute Inflammation and Cardiac Repair

Heart tissues were collected at the indicated time points for immunofluorescence analysis. At 24 h after reperfusion, frozen or paraffin‐embedded heart sections were stained to assess oxidative stress and inflammatory activation, including DHE and MitoSOX staining for ROS detection and immunofluorescence staining for S100a9, citrullinated histone H3 (CitH3), and myeloperoxidase (MPO). At 4 weeks after surgery, cardiac sections were stained with primary antibodies against α‐actinin, Cx43, CD31, and α‐smooth muscle actin (α‐SMA) to evaluate cardiomyocyte structural organization, gap‐junction remodeling, angiogenesis, and vessel maturation. Fluorescence images were acquired using a Zeiss Celldiscoverer 7 microscope. Detailed procedures for Evans blue/TTC staining, plasma biomarker detection, Masson's trichrome staining, and H&E staining are provided in the Supporting Information.

### 4.13 Statistical Analysis

All data are presented as mean ± standard deviation. Sample sizes are indicated in the figure legends. Statistical analyses were performed using GraphPad Prism software. For comparisons between two groups, an unpaired Student's *t*‐test was used. For comparisons among multiple groups, one‐way ANOVA followed by Tukey's post hoc test was performed. For echocardiographic parameters measured over time, two‐way repeated‐measures ANOVA was used where appropriate. Categorical data, such as VT inducibility, were analyzed using Fisher's exact test or the chi‐square test where appropriate. A value of *p* < 0.05 was considered statistically significant.

## Conflicts of Interest

The authors declare no conflicts of interest.

## Supporting information




**Supporting File 1**: advs76421‐sup‐0001‐SuppMat.docx.

**Supporting File 2**: advs76421‐sup‐0002‐VideoS1‐S2.zip.

## Data Availability

The data that support the findings of this study are available on request from the corresponding author. The data are not publicly available due to privacy or ethical restrictions.
