# Supplementary material for: Celastrol‐Loaded Conductive Hydrogel Mitigates Myocardial Ischemia‐Reperfusion Injury and Restores Electrophysiological Function
Source: Adv Sci (Weinh). 2026 Jul 10;13(54):e24201. doi: 10.1002/advs.202524201 (PMC13353178; doi:10.1002/advs.202524201)
Supplement: Supplementary file 1 [file ADVS-13-e24201-s001.docx]

**Celastrol-Loaded Conductive Hydrogel Mitigates Myocardial Ischemia–Reperfusion Injury and Restores Electrophysiological Function**

*Shixin Wang ^1, †^,* *Shaojie Chen ^1, †^, Chengzong Li ^2, †^, Hongyi Cheng ^1^, Jincheng Jiao ^1^, Xiafeng Peng ^1^, Yike Zhang ^1^, Wei Sun ^1, *^, Feng Zhang ^3, *^, Chang Cui ^1, *^, Minglong Chen ^1,4^*

^1^Department of Cardiology, The First Affiliated Hospital with Nanjing Medical University, Nanjing 210029, P.R. China

^2^Department of Cardiology, The Affiliated Hospital of Xuzhou Medical University, Xuzhou 221002, P.R. China

^3^Jiangsu Clinical Medicine Research Institute, the First Afﬁliated Hospital with Nanjing Medical University, Nanjing 210029, P.R. China

^4^Department of Cardiology, The Affiliated Taizhou People’s Hospital of Nanjing Medical University, Taizhou School of Clinical Medicine, Nanjing Medical University, Taizhou 225300, P.R. China

*Corresponding author

E-mail addresses: weisun7919@njmu.edu.cn (W. Sun); zhangfeng@njmu.edu.cn (F. Zhang); cuichang@njmu.edu.cn (C. Cui);

^†^ These authors contributed equally to this work.

**1. Materials**

Pluronic F127 diacrylate (F127DA), Gelatin methacryloyl (GelMA), and Lithium Phenyl-2,4,6-trimethylbenzoylphosphinate (LAP) were obtained from Engineering for Life (EFL, Zhejiang, China). Poly(3,4-ethylenedioxythiophene): poly (styrenesulfonate) (PEDOT:PSS) was purchased from Sigma-Aldrich (USA). Celastrol (CLT) was purchased from MedChemExpress (MCE, USA). The Calcein-AM/PI double staining kit, 2′,7′-dichlorodihydrofluorescein diacetate (DCFH-DA) staining kit, and MitoSOX™ Red mitochondrial superoxide indicator kit were obtained from Beyotime (Shanghai, China). Dihydroethidium (DHE) was purchased from Beijing Chemical Reagent Company (Beijing, China). The Cell Counting Kit-8 (CCK-8) for cell proliferation/cytotoxicity assays was purchased from Dojindo Laboratories (Tokyo, Japan). CK-MB ELISA kit (JL13014) and cTnI ELISA kit (JL12296) were purchased from Jianglai Biotechnology, Shanghai, China. All reagents were of analytical grade and used directly without further purification.

**2. Methods**

**2.1. Electrical Conductivity Measurement of Hydrogels**

The electrical conductivity of the hydrogels was measured using a four-point probe method. Hydrogels were prepared in cylindrical molds and fully crosslinked before testing. Conductivity was first screened in hydrogels containing different PEDOT:PSS contents to determine an appropriate formulation, and then compared among representative groups including F, G, FG, FGP, and FGPC. Data were expressed as conductivity values in S/cm. Three independently prepared samples were measured for each group (n = 3).

**2.2. In Vitro Degradation and Swelling of FGPC Hydrogel**

Crosslinked FGPC hydrogels of identical size were prepared for in vitro degradation and swelling assays. For the degradation assay, freshly crosslinked hydrogels were gently blotted with filter paper to remove excess surface liquid, and the initial wet weight was recorded as $W_{0}$. The hydrogels were then immersed in PBS and incubated at 37 °C. At predetermined time points, the samples were collected, gently rinsed with PBS, blotted to remove surface liquid, and weighed again. The wet weight at each time point was recorded as $W_{t}$. The remaining mass percentage was calculated using the following equation:

$$\text{Re}\text{maining mass}(\%)=\frac{W_{t}}{W_{0}}\times100\%$$

where $W_{0}$ is the initial wet weight of the hydrogel and $W_{t}$ is the wet weight at each time point.

For the swelling assay, freshly crosslinked FGPC hydrogels of identical size were gently blotted to remove excess surface liquid, and the initial wet weight was recorded as $W_{0}$. The hydrogels were immersed in PBS at 37 °C. At predetermined time points, the samples were removed, gently blotted with filter paper, and immediately weighed. The swollen weight at each time point was recorded as $W_{t}$. The relative swelling ratio was calculated using the following equation:

$$\text{Relative swelling ratio}(\%)=\frac{W_{t}-W_{0}}{W_{0}}\times100\%$$

where $W_{0}$is the initial wet weight of the hydrogel and $W_{t}$is the wet weight after swelling for the indicated time. Three independently prepared samples were analyzed at each time point ($n=3$).

**2.3. In Vivo Degradation of the FGPC Hydrogel**

The in vivo degradation profile of the FGPC hydrogel was evaluated via a subcutaneous implantation model. Rats were anesthetized with isoflurane (2-3% in oxygen), and the thoracic region skin was shaved and disinfected. FGPC pre-gel solution (200 μL) was injected subcutaneously into the thoracic region and crosslinked in situ using 405 nm blue light irradiation (~10 mW cm⁻²) for 60 s. The implantation sites were monitored daily for signs of infection or inflammation. At predetermined time points (days 1, 3, 7, and 14), the rats were euthanized, and the implantation sites were surgically exposed to visually inspect and document the residual hydrogel. The degradation was quantified based on the remaining gel volume or area, and the time point at which the hydrogel was almost completely absorbed was recorded.

**2.4. In Vivo Fluorescence Imaging of Cy5-CLT Retention**

Male Sprague-Dawley rats weighing approximately 220 g were anesthetized with 2% isoflurane, intubated, and mechanically ventilated. Myocardial ischemia was induced by transient ligation of the left anterior descending coronary artery for 30 min, followed by reperfusion. For fluorescence tracking, Cy5-labeled CLT (Cy5-CLT) was used for fluorescence tracking. Immediately after reperfusion, rats were randomly assigned to two groups: the FGPC group, in which Cy5-CLT was encapsulated in the FGPC pre-gel solution and locally applied onto the epicardial surface of the peri-infarct myocardium, and the free Cy5-CLT group, in which an equivalent dose of Cy5-CLT in the same vehicle was locally applied at the same site. In vivo fluorescence imaging was performed at days 1, 3, 7, and 14 after treatment using an IVIS Spectrum imaging system. At each time point, rats were anesthetized, and fluorescence signals from the thoracic region were acquired using identical imaging parameters.

**2.5. Quantitative Real-Time PCR**

Total RNA was extracted from myocardial infarct border-zone tissues using TRIzol reagent according to the manufacturer’s instructions. RNA concentration and purity were assessed using a NanoDrop spectrophotometer. Complementary DNA was synthesized using a reverse transcription kit. Quantitative real-time PCR was performed using SYBR Green qPCR Master Mix on a real-time PCR system. The relative mRNA expression levels of S100a8, S100a9, Il6, and Cxcl1 were normalized to GAPDH and calculated using the 2^-ΔΔCt method. Six biologically independent samples were included in each group (n = 6).

**2.6. Evans Blue/TTC Staining**

At 24 h after reperfusion, the LAD was re-ligated at the original ligation site, and Evans blue dye was injected to delineate the non-ischemic myocardium. Hearts were rapidly excised, rinsed with cold saline, briefly frozen, and sectioned into serial transverse slices. The slices were incubated with TTC solution at 37 °C and then fixed in 4% paraformaldehyde. TTC-negative pale regions were defined as infarcted myocardium, whereas TTC-positive red regions represented viable myocardium. Infarct size was quantified using ImageJ and expressed as the percentage of infarcted area relative to the left ventricular area (INF/LV).

**2.7. Measurement of Plasma CK-MB and cTnI Levels**

For comparison of acute myocardial injury, Free CLT was administered intraperitoneally at 1 mg kg⁻¹ immediately after reperfusion, whereas FGPC was locally applied to the epicardial surface immediately after reperfusion. At 24 h after reperfusion, blood samples were collected and centrifuged to obtain plasma. Plasma levels of CK-MB and cTnI were measured using commercial ELISA kits, including CK-MB ELISA kit (JL13014, Jianglai Biotechnology, Shanghai, China) and cTnI ELISA kit (JL12296, Jianglai Biotechnology, Shanghai, China), according to the manufacturer’s instructions. Concentrations were calculated based on standard curves.

**2.8. Masson’s Trichrome and H&E Staining**

At 4 weeks after surgery, hearts and major organs were harvested, fixed in 4% paraformaldehyde, embedded in paraffin, and sectioned. Masson’s trichrome staining was performed on heart sections to evaluate myocardial fibrosis. H&E staining was performed on major organs, including the liver, kidney, heart, and testis, to assess potential off-target toxicity. Images were acquired using a light microscope, and fibrotic area was quantified using ImageJ.

**2.9. Immunofluorescence Staining and Antibody Information**

Heart tissues or cell samples were fixed, embedded in paraffin or optimal cutting temperature compound where appropriate, and sectioned for immunofluorescence analysis. Samples were incubated overnight at 4 °C with primary antibodies, including mouse anti-α-actinin (A7732), rabbit anti-Cx43 (3512S), rabbit anti-S100a9 (26992-1-AP), rabbit anti-citrullinated histone H3 (CitH3; ab281584), mouse anti-myeloperoxidase (MPO; ab90810), rabbit anti-CD31 (28083-1-AP), and mouse anti-α-smooth muscle actin (α-SMA; 67735-1-Ig). After washing, samples were incubated with appropriate Alexa Fluor-conjugated secondary antibodies, including Alexa Fluor 488 goat anti-mouse IgG (ab150113), Alexa Fluor 488 goat anti-rabbit IgG (ab150077), Alexa Fluor 594 goat anti-mouse IgG (ab150116), and Alexa Fluor 594 goat anti-rabbit IgG (ab150080). Nuclei were counterstained with Hoechst or DAPI before fluorescence imaging.

**3. Results**

**Figure S1.** Concentration-window validation of celastrol under oxidative stress. (a) Cell viability of AC16 cells treated with different concentrations of celastrol under H₂O₂-induced oxidative stress, as determined by CCK-8 assay. (b) Relative ROS levels in AC16 cells treated with different concentrations of celastrol under H₂O₂-induced oxidative stress. ROS levels were normalized to the corresponding cell viability values. Data are presented as mean ± SD (n = 5). Statistical significance was determined by one-way ANOVA followed by Tukey’s post hoc test. (*p < 0.05, **p < 0.01, ***p < 0.001; ns, not significant.)

**Table S1.** The compositions of various hydrogels.

| Hydrogels | F127DA (g) | GelMA (g) | PEDOT:PSS (1.5 wt%) | LAP (0.5 wt%) | Celastrol (10 mM) | PBS |
| --- | --- | --- | --- | --- | --- | --- |
| F127DA (F) | 0.5 | - | - | 2.5 mL | - | 2.5 mL |
| GelMA (G) | - | 0.375 | - | 2.5 mL | - | 2.5 mL |
| F127DA+GelMA (FG) | 0.5 | 0.375 | - | 2.5 mL | - | 2.5 mL |
| F127DA+GelMA+PEDOT:PSS (FGP) | 0.5 | 0.375 | 0.333 mL | 2.5 mL | - | 2.167 mL |
| F127DA+GelMA+PEDOT:PSS+ Celastrol (FGPC) | 0.5 | 0.375 | 0.333 mL | 2.5 mL | 1 μL | 2.167 mL |

**Figure S2.** Encapsulation and release characterization of Cy5-CLT. (a) Representative photographs showing the appearance of F127DA solution before and after Cy5-CLT incorporation. (b) Standard curve showing the relationship between Cy5-CLT concentration and fluorescence intensity. (c) Representative photographs of Cy5-CLT release from FGPC hydrogel in release medium at indicated time points.

**Table S2.** Kinetic fitting of Cy5-CLT release from FGPC hydrogel.

| Model | Equation | R² |
| --- | --- | --- |
| Zero-order | Qt = 0.4235t + 20.1587 | 0.6636 |
| First-order | ln (100 − Qt) = 4.3729 − 0.006422t | 0.7586 |
| Higuchi | Qt = 4.6676t^1/2 + 11.9857 | 0.8206 |
| Korsmeyer–Peppas | Mt/M∞ = 0.0702t^0.5311 | 0.6823 |

**Note:** Qt represents the cumulative release percentage of Cy5-CLT at time $t$, and $M_{t}/M_{\infty}$represents the fractional drug release at time $t$. Among the tested models, the Higuchi model showed the highest $R^{2}$value, suggesting that diffusion contributed substantially to Cy5-CLT release from the FGPC hydrogel.

**Figure S3.** In vitro degradation of FGPC hydrogel. Remaining mass of FGPC hydrogel after incubation in PBS at 37 °C for the indicated time points. Data are presented as mean ± SD (n = 3).

**Figure S4.** In vitro swelling of FGPC hydrogel. Relative swelling ratio of FGPC hydrogel after incubation in PBS at 37 °C for the indicated time points. Data are presented as mean ± SD (n = 3).

**Figure S5.** Electrical conductivity of different hydrogels. The electrical conductivity of hydrogels in different groups was measured using a four-point probe. Data are presented as mean ± SD (n = 3). Statistical significance was determined by one-way ANOVA followed by Tukey’s post hoc test. (*p < 0.05, **p < 0.01, ***p < 0.001; ns, not significant).

**Figure S6.** qPCR validation of representative inflammation-related genes identified by RNA sequencing. Relative mRNA expression levels of (a) *S100a8*, (b) *S100a9*, (c) *Il6*, and (d) *Cxcl1* in myocardial tissues from the Sham, I/R, FGP, and FGPC groups, as determined by qPCR. Data are presented as mean ± SD (n = 6). Statistical significance was determined by one-way ANOVA followed by Tukey’s post hoc test. (*p < 0.05, **p < 0.01, ***p < 0.001; ns, not significant.)

**Figure S7.** Pharmacokinetics and biodistribution of CLT in vivo. (a) Schematic illustration of CLT administration and sampling schedule. Free CLT was administered intraperitoneally once daily as the systemic delivery control, whereas CLT-loaded FGPC was locally applied to the epicardial surface after reperfusion. Blood and major organs were collected at the indicated time points for LC–MS/MS analysis. (b) Plasma concentration–time profile of free CLT after intraperitoneal administration. (c) Plasma concentration–time profile of CLT after local FGPC delivery. (d) Tissue distribution of free CLT in the heart, liver, spleen, lung, and kidney at 1, 3, 7, and 14 days after administration. (e) Tissue distribution of FGPC-delivered CLT in the heart, liver, spleen, lung, and kidney at 1, 3, 7, and 14 days after administration. The dashed line indicates the lower limit of quantification. Data are presented as mean ± SD (n = 3).

**Figure S8.** Comparison of acute myocardial injury between free CLT and FGPC treatment after ischemia–reperfusion. (a) Representative Evans blue/TTC-stained heart sections from the Sham, I/R, Free CLT, and FGPC groups. (b) Quantitative analysis of infarct size expressed as INF/LV. (c, d) Plasma levels of CK-MB (c) and cTnI (d) at 24 h after reperfusion, as measured by ELISA. Data are presented as mean ± SD (n = 6). Statistical significance was determined by one-way ANOVA followed by Tukey’s post hoc test. (*p < 0.05, **p < 0.01, ***p < 0.001; ns, not significant.)

**Figure S9.** Surgical validation and echocardiographic assessment. (a) Representative ECG traces before LAD occlusion, during occlusion (ST-segment elevation), and after reperfusion (ST-segment regression). (b) Representative H&E-stained heart sections from different groups at 4 weeks after surgery. Scale bar, 2 mm. (c) Representative macroscopic images of epicardial hydrogel application and in situ photo-crosslinking. (d) Quantification of left ventricular wall thickness at 4 weeks after surgery, including LVAW;s, LVAW;d, LVPW;s, and LVPW;d. (e-g) Quantification of LVEF at baseline (e), 1 week (f), and 2 weeks (g) after surgery. Data are presented as mean ± SD (n = 8). Statistical significance was determined by one-way ANOVA followed by Tukey’s post hoc test. (*p < 0.05, **p < 0.01, ***p < 0.001; ns, not significant.)

**Figure S10.** Representative intracardiac traces during S1–S2 stimulation. Representative intracardiac electrogram traces recorded during programmed electrical stimulation in the (a) Sham, (b) I/R, (c) FGP, and (d) FGPC groups. S1 indicates a train of basic stimuli, and S2 indicates a single extrastimulus. VT, ventricular tachycardia; VF, ventricular fibrillation.

**Figure S11.** Biosafety evaluation of FGPC hydrogel. (a) Representative H&E-stained images of major organs, including the heart, liver, kidney, and testis, at 4 weeks after surgery. Scale bars, 100 μm. (b-e) Serum biochemical analysis of liver and kidney function, including ALT (b), AST (c), CREA (d), and BUN (e). (f) Percentage of circulating neutrophils among total white blood cells. Data are presented as mean ± SD (n = 6). Statistical significance was determined by one-way ANOVA followed by Tukey’s post hoc test. (*p < 0.05, **p < 0.01, ***p < 0.001; ns, not significant.)
